# Antioxidant-mediated suppression of ferroptosis in *Pyricularia oryzae*: a novel approach to rice blast management for sustainable rice production

**DOI:** 10.3389/fpls.2024.1520688

**Published:** 2024-12-20

**Authors:** Mattia Santoni, Junior Bernardo Molina-Hernandez, Andrea Kunova, Paolo Cortesi, Barbara Brunetti, Pietro Rocculi, Michael S. Christodoulou, Francesca Danesi

**Affiliations:** ^1^ Department of Agricultural and Food Sciences (DISTAL), University of Bologna, Cesena, Italy; ^2^ Department of Food, Environmental and Nutritional Sciences (DeFENS), University of Milan, Milan, Italy; ^3^ Interdepartmental Centre for Industrial Agri-Food Research (CIRI), University of Bologna, Cesena, Italy

**Keywords:** antioxidants, ferroptosis, *Magnaporthe oryzae*, peroxidation, plant-pathogen interactions, *Pyricularia oryzae*, reactive oxygen species, rice blast disease

## Abstract

Ferroptosis, an iron-dependent form of regulated cell death, has recently emerged as a crucial process in the pathogenesis of *Pyricularia oryzae*, the causal agent of the devastating rice blast disease, which causes billions of dollars in annual losses. This mini review explores the potential of antioxidants in suppressing ferroptosis in *P. oryzae* to promote sustainable rice production, with significant implications for global food security and nutrition. We critically analyze the current literature on the mechanisms of ferroptosis in *P. oryzae*, including iron metabolism and lipid peroxidation, the role of different antioxidants in inhibiting this cell death pathway, and the potential applications of antioxidant-based strategies for the management of rice blast disease. Recent discoveries, such as the efficacy of the natural flavonoid tangeretin in inhibiting fungal ferroptosis by interfering with the accumulation of iron and reactive oxygen species, highlight the promise of natural and nature-inspired compounds for disease management. The use of antioxidants to modulate ferroptosis in *P. oryzae* offers several advantages over traditional fungicide-based approaches, including improved safety, sustainability, and potential nutritional benefits through antioxidant-enriched rice varieties. However, challenges such as optimizing delivery methods, managing potential resistance, and ensuring efficacy under different environmental conditions need to be addressed. To achieve these goals, future research should focus on identifying the most effective antioxidant compounds, exploring synergistic combinations, and developing sustainable application methods.

## 1 Introduction

Rice, the most important staple food for over half of the global population, faces a major threat from blast disease caused by *Pyricularia oryzae* (teleomorph *Magnaporthe oryzae*). This ascomycete filamentous fungus significantly impacts global rice production and food security (Islam et al., 2023), leading to 10–30% yield losses (Dean et al., 2012), with severe outbreaks causing up to 50% crop loss (Scheuermann et al., 2012; Nalley et al., 2016). *P. oryzae* infects various parts of rice plants (leaves, stems, nodes, and panicles), causing widespread damage. Different lineages are able to infect also other important cereal crops like millet, barley, and wheat (Wilson, 2021). Of particular global concern is the *P. oryzae* pathotype *Triticum*, which causes wheat blast—a devastating disease that impacts wheat, another major staple crop (Castroagudin et al., 2016). Wheat blast has already led to significant crop losses in South America and South Asia, with the potential to spread further. This pathotype poses a serious threat to global food security due to its adaptability and resistance to common fungicides (Cruz and Valent, 2017; Ceresini et al., 2018). Ongoing research focuses on early detection methods, molecular markers for specific pathotype identification, and the development of resistant wheat varieties to mitigate the impact of this dangerous pathogen (Ikeda et al., 2024).

The economic impact of blast is substantial, with annual damage estimated at $70 billion (Scheuermann et al., 2012; Valent, 2021). Despite ongoing research, effective long-term solutions remain elusive, and climate change alongside with increasing pathogen resistance urge for innovative management approaches (Singh and Maurya, 2021; Singh et al., 2023).

Recent advances in understanding the pathogenesis of *P. oryzae* have revealed the role of ferroptosis, an iron-dependent regulated cell death, in the infection process (Shen et al., 2020). Characterized by lipid peroxide and iron-dependent reactive oxygen species (ROS) accumulation, ferroptosis is crucial for the development of infection structures and disease progression (Kou et al., 2019; Shen et al., 2020, 2023). Evidence suggests that iron and lipid peroxidation are necessary for ferroptosis spread, and involve a signal that propagates upstream of cell rupture (Riegman et al., 2020). This discovery has prompted research into antioxidants as a novel disease control strategy. Antioxidants have demonstrated the ability to suppress ferroptosis in various biological systems (Ge et al., 2021; Zhang et al., 2021; Rizzardi et al., 2022; Zhang et al., 2024), sparking interest in their potential to interrupt the infection cycle of *P. oryzae* and enhance rice plant resistance (Liu and Zhang, 2022).

This mini review summarizes current knowledge of ferroptosis in pathogenesis of *P. oryzae* and the potential of antioxidants in suppressing this process. Our focus is specifically on antioxidant-based approaches to suppress ferroptosis in *P. oryzae*, rather than strategies such as biological control, breeding for resistant cultivars, or genetic engineering. We examine recent advances, discuss antioxidant interventions, and explore implications for sustainable management of rice blast. We also highlight controversies, identify research gaps, and propose future directions, aiming to provide a concise overview of how targeting ferroptosis through antioxidant strategies could contribute to more effective and environmentally friendly approaches to manage rice blast disease, supporting global food security and sustainable agriculture.

## 2 Ferroptosis: mechanisms and significance in *Pyricularia oryzae*

The hallmarks of ferroptosis in *P. oryzae* are the accumulation of lipid peroxides, elevated levels of intracellular ferric iron (Fe ^3+^), and the consequent generation of ROS (Liu et al., 2024). Researchers have identified key players in this process, highlighting the critical role of iron metabolism regulated by the transcription factor Fep1 (Kou et al., 2019; Liu and Zhang, 2022). Abdul et al. (2018) showed that cell death is characterized by membrane damage caused by the accumulation of lipid peroxides to lethal concentrations due to the oxidation of polyunsaturated fatty acids in membrane phospholipids, which is essential for ferroptotic cell death, underlining the importance of membrane integrity. Recent research has also highlighted the role of calcium signaling with high Ca^2+^ levels in ROS-dependent cell death due to an imbalance in cellular redox status, such as in ferroptosis (Molina-Hernandez et al., 2022).

In *P. oryzae*, ROS regulation plays important roles in both development and virulence. ROS generation has been linked to the NADPH oxidase (NOX) complex. A pioneering work by Egan et al. (2007) showed that NOX1 and NOX2 are important sources of ROS during appressorium development and ferroptosis, thus representing potential targets for possible control strategies.

Shen and colleagues (Shen et al., 2020; Shen and Naqvi, 2021; Shen et al., 2023; Shen and Naqvi, 2024) have shown that ferroptosis is essential for appressorium maturation, successful rice cell penetration, and rice tissue colonization. Modulating ferroptosis can significantly affect the virulence of *P. oryzae*, suggesting potential disease control avenues.

Despite these advances, controversy remains. Stockwell et al. (2017) highlighted the need for precise molecular markers to differentiate ferroptosis from other cell death forms. The role of ferroptosis in *P. oryzae* strains infecting non-rice hosts is still largely unexplored (Shen and Naqvi, 2024).

Environmental influences on ferroptosis represent another area of uncertainty. Studies have questioned how temperature (Onaga et al., 2017), humidity (Qiu et al., 2022), and drought stress (Bidzinski et al., 2016) might influence the pathogenic process and virulence under changing climatic conditions. However, the direct effects of these factors on ferroptosis in *P. oryzae* are not yet fully understood.

## 3 Natural and synthetic compounds and their role in suppressing ferroptosis in *Pyricularia oryzae*

**Figure 1:**
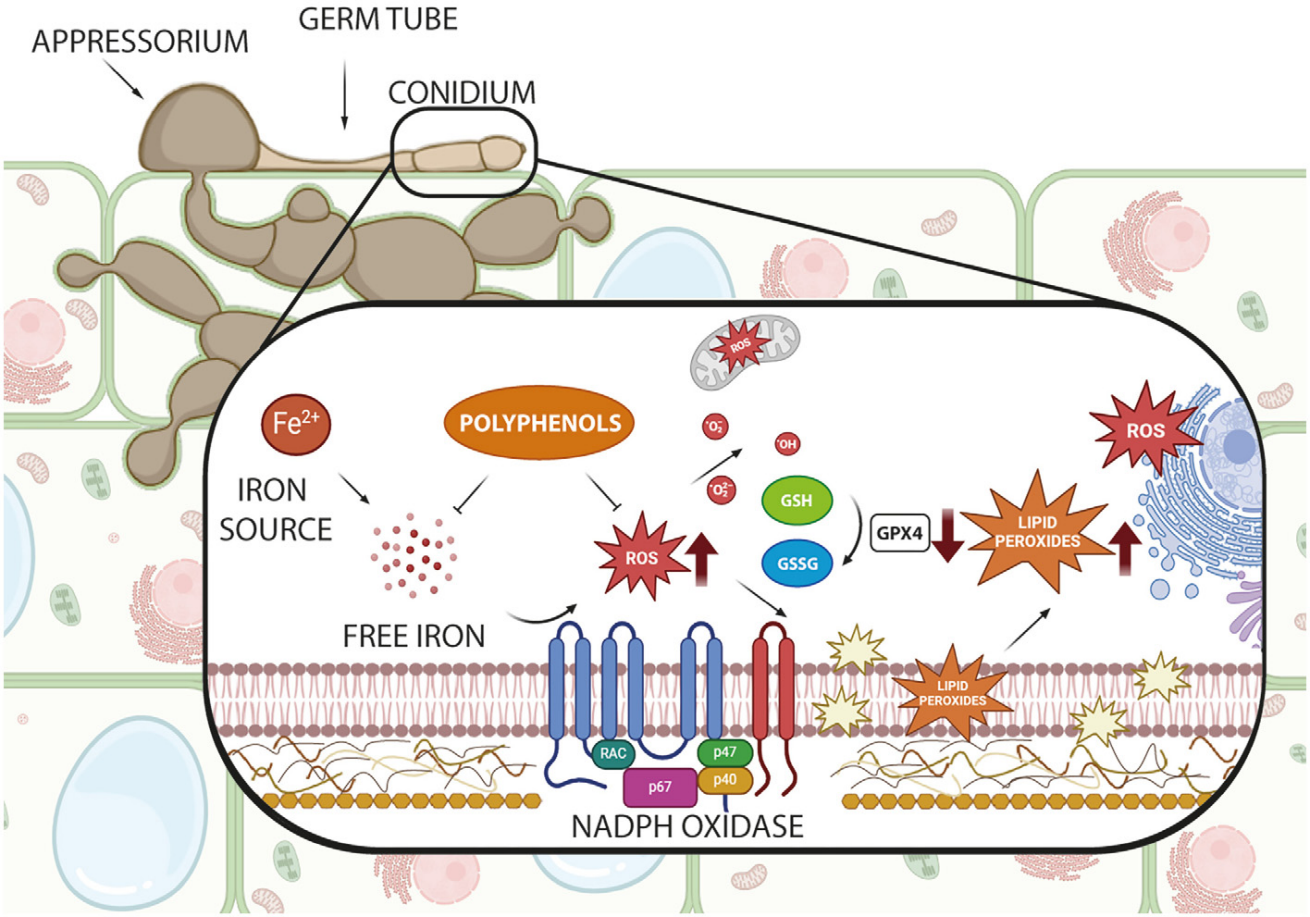
Schematic representation of the ferroptosis pathway in *Pyricularia oryzae* and potential antioxidant intervention points. The figure shows key cellular components involved in ferroptosis, including iron sources, NADPH oxidase, reactive oxygen species (ROS), lipid peroxides, and the glutathione (GSH)/glutathione disulfide (GSSG), part of glutathione peroxidase 4 (GPX4) system. Created with BioRender.com.

Natural and synthetic compounds have shown effectiveness in modulating ferroptosis, offering promising possibilities for controlling *P. oryzae* infections (Sies et al., 2017). While the role of ferroptosis in *P. oryzae* pathogenicity has been established (Shen et al., 2020), the specific effects of inhibitory compounds—whether natural, nature-inspired, or synthetic—on this cell death pathway remain under investigation. **Figure 1** illustrates the key molecular components involved and highlights potential intervention targets. Natural antioxidants, particularly glutathione and (poly)phenolics (PCs), along with nature-inspired and synthetic inhibitors, have emerged as promising agents for suppressing this pathogenic process in *P. oryzae*.

### 3.1 Glutathione and related systems

**Figure 2:**
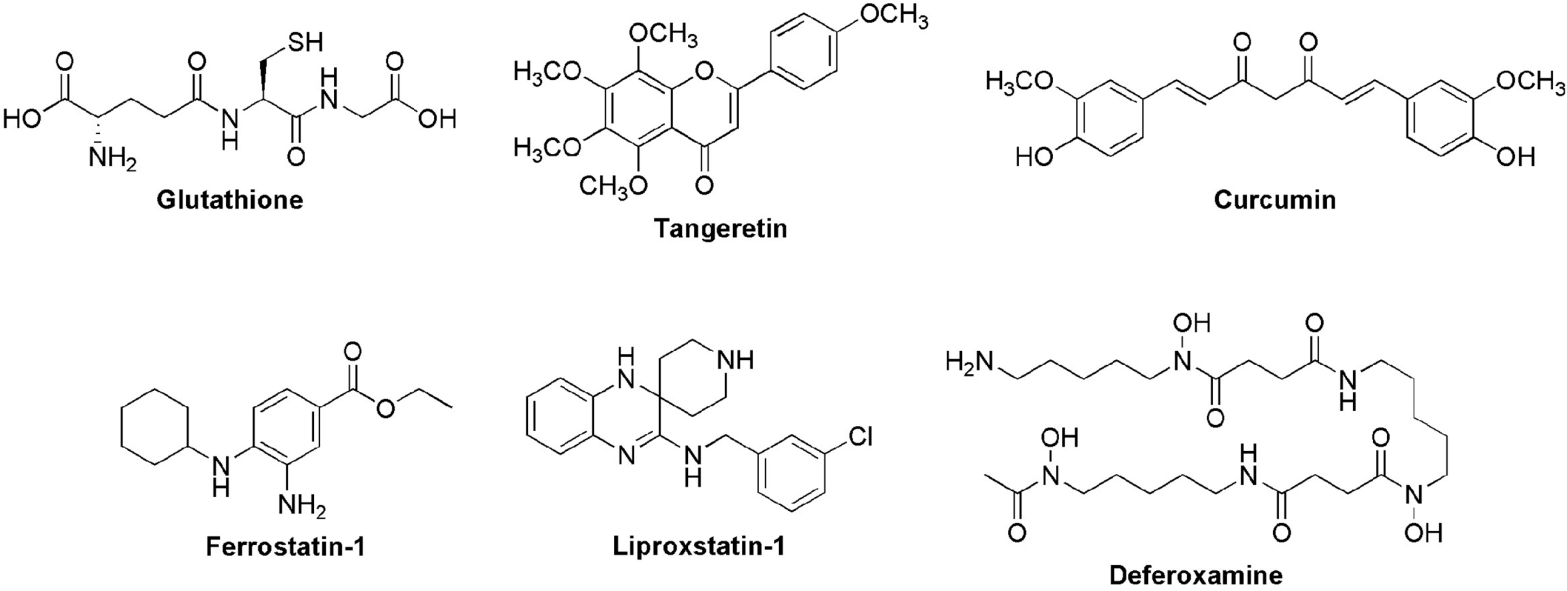
Molecular structures of antioxidants (glutathione, tangeretin and curcumin), synthetic inhibitors of ferroptosis (ferrostatin-1 and liproxstatin-1), and iron chelators (deferoxamine) involved in modulating iron-dependent cell death in *P. oryzae*.

Glutathione (GSH), a crucial cellular antioxidant (**Figure 2**), plays a vital role in regulating ferroptosis. Stockwell and colleagues (2017) established that GSH is essential for maintaining cellular redox balance and detoxifying lipid hydroperoxides through glutathione peroxidase 4. Fernandez and Wilson (2014) demonstrated the importance of glutathione-related systems for *P. oryzae* virulence in rice blast disease. Huang et al. (2011) identified the MoHYR1 gene in *P. oryzae*, encoding a protein with a GPX domain that utilizes GSH to detoxify ROS. Deletion of MoHYR1 increased sensitivity to H_2_O_2_ and reduced virulence, linking GSH-dependent mechanisms to the pathogen’s ability to overcome host defenses.

Samalova et al. (2014) showed that *P. oryzae* maintains a highly reduced cytoplasmic glutathione pool during infection, with only slight shifts in oxidation during development. This tight regulation of GSH redox state, coupled with the fungus’s extreme resistance to external H_2_O_2_ exposure, underscores robust antioxidant defenses of *P. oryzae*. In fact, more recent studies with *P. oryzae* have demonstrated that rice produces H_2_O_2_ shortly after inoculation with a virulent strain of *P. oryzae* (Chi et al., 2009; Kato et al., 2009). Dangol et al. (2019) showed that glutathione depletion, induced by erastin, a small antitumor agent, leads to iron- and ROS-dependent ferroptotic cell death in rice cells during *P. oryzae* infection, highlighting the interplay among glutathione, iron, and ROS in plant-pathogen interactions.

### 3.2 Tangeretin and other (poly)phenolics

PCs have shown promise as antioxidants and potential suppressors of oxidative stress in various biological systems (Quideau et al., 2011). Curcumin (**Figure 2**), for instance, demonstrated antifungal properties against plant pathogens (Hu et al., 2017), suggesting potential for further investigation in *P. oryzae* ferroptosis through suppression of iron accumulation.

Recent research has expanded our understanding of PCs’ role in plant-fungal interactions. Moin et al. (2024) conducted an *in silico* study suggesting that certain flavonoids may influence pathogenicity of *P. oryzae*. These results support the potential of PCs in plant-fungal interactions (Shalaby and Horwitz, 2015). In 2021, Liang et al. (2021) highlighted the potential of PCs in suppressing ferroptosis in *P. oryzae*. Notably, tangeretin, a flavonoid from citrus peels (**Figure 2**), effectively inhibits fungal ferroptosis and suppresses rice blast disease by impairing iron and ROS accumulation and suppressing lipid peroxidation in *P. oryzae* conidia, which are crucial for appressorium formation and subsequent pathogenesis.

These findings on natural antioxidants, particularly flavonoids like tangeretin and other PCs, not only demonstrate their direct potential in suppressing ferroptosis in *P. oryzae*, but also provide valuable structural and functional insights that could serve as templates for the design and synthesis of novel, nature-inspired molecules with enhanced efficacy and specificity against rice blast disease. Additionally, other PCs in rice, such as hydroxybenzoic and hydroxycinnamic acids, play a vital role in plant defense by enhancing structural integrity, acting as direct antimicrobial agents, and regulating hypersensitive responses during biotic stress (see **Supplementary Table 1**).

### 3.3 Other ferroptosis inhibitors and iron chelators

While ferroptosis inhibitors like ferrostatin-1 and liproxstatin-1, whose structures are shown in **Figure 2**, have shown efficacy in mammalian systems (Dixon et al., 2012; Friedmann Angeli et al., 2014; Miotto et al., 2020; Scarpellini et al., 2023), their effects on *P. oryzae* are only now being explored. In recent years, research has begun to investigate the potential of other ferroptosis inhibitors (*e.g.*, ferrostatin-1 and deferoxamine (DFO) - structures shown in **Figure 2**) in plant-pathogen interactions (Dangol et al., 2019), opening new possibilities for chemical control strategies of *P. oryzae*. For example, Christodoulou et al. (2024) demonstrated that DFO inhibits appressorium formation, likely through its chelating ability. The authors speculate that fungal cells might uptake DFO via specific transport systems such as proton symporters, actively transporting the compound based on extracellular iron concentrations. This targeted mechanism could significantly disrupt early conidial development, thereby reducing the virulence of the rice blast fungus.

## 4 Implications of antioxidant-based strategies in rice blast control for human nutrition

The use of antioxidants to manage rice blast disease has a significant impact on global nutrition and food security. Rice provides essential nutrients for billions of people, especially in developing countries (Muthayya et al., 2014). Ensuring sustainable rice production and minimizing yield losses due to diseases such as rice blast are critical to preventing hunger and malnutrition worldwide (Nalley et al., 2016).

Agronomical approaches to enhance endogenous antioxidant levels in rice could provide dual benefits for both disease resistance and nutritional value. For GSH content, studies have shown that appropriate timing of nitrogen fertilization and water management practices can optimize GSH biosynthesis pathways and maintain cellular redox homeostasis through regulation of the GSH/GSSG ratio (Hasanuzzaman et al., 2017; Cimini et al., 2022). For PCs, several agronomical practices have been shown to modulate their accumulation in rice. For example, targeted stress conditions during grain development can enhance phenylpropanoid pathway activity and the resulting PC content (Yang et al., 2024). These agronomic interventions could be integrated with new approaches based on natural antioxidants, such as flavonoids like tangeretin which effectively inhibits fungal ferroptosis (Liang et al., 2021), into existing rice cultivation systems to enhance both disease resistance and nutritional value of rice crops (Pang et al., 2018).

Recent clinical studies have demonstrated that consumption of pigmented rice, particularly rich in PCs, mainly ferulic acid and anthocyanins, can improve antioxidant status (Mendoza-Sarmiento et al., 2023), while dietary supplements like curcumin can enhance plasma GSH levels, leading to improved cardiometabolic health through reduced oxidative stress and inflammation (Dludla et al., 2023).

## 5 Challenges and future directions

While antioxidants show great promise in suppressing ferroptosis in *P. oryzae*, several challenges remain. The specificity of antifungal action, potential off-target effects, and the development of fungal resistance are concerns that need to be addressed (Fisher et al., 2018). Moreover, the translation of laboratory findings to field applications presents logistical and regulatory hurdles (Hollomon, 2015). Future research should focus on identifying natural antioxidants that can effectively suppress ferroptosis in *P. oryzae* while being safe and bioavailable for human consumption (Goufo and Trindade, 2017), including optimizing delivery methods and exploring synergistic combinations with other antifungals. Investigating the potential of enhancing endogenous antioxidant systems in rice plants represents an exciting avenue for increasing resistance to *P. oryzae* infection (Yang et al., 2024). Additionally, research should explore how chitin-derived signals from fungal cell walls, which act as defense elicitors in rice (Kaku et al., 2006), might interact with iron and ROS-dependent pathways during *P. oryzae* infection. This multifaceted approach requires interdisciplinary collaboration among plant pathologists, nutritionists, agronomists, and food scientists to fully realize the potential of antioxidant-based strategies in both rice blast control and nutrition enhancement.

## 6 Conclusion

The rice blast disease, caused by *P. oryzae*, remains a significant threat to global rice production and food security. This mini review has explored the promising strategy of using antioxidants to suppress ferroptosis in *P. oryzae* for controlling this devastating disease. Recent discoveries, such as the efficacy of tangeretin in inhibiting fungal ferroptosis, highlight the potential of natural and nature-inspired compounds in rice blast management. These findings offer alternatives to traditional fungicides and opportunities to enhance rice’s nutritional value through antioxidant enrichment.

Antioxidant-based approaches present several advantages, including improved safety and environmental friendliness compared to synthetic fungicides. However, challenges remain in optimizing delivery methods, addressing potential resistance, and ensuring efficacy in diverse conditions. Future research should focus on identifying effective antioxidant compounds, exploring synergistic combinations, and developing sustainable application methods. From a nutritional perspective, this approach offers possibilities for enhancing both rice resilience and its nutritional quality.

## References

[B1] Abdul W. Aliyu S. R. Lin L. Sekete M. Chen X. Otieno F. J.. (2018). Family-four aldehyde dehydrogenases play an indispensable role in the pathogenesis of *Magnaporthe oryzae* . Front. Plant Sci. 9. doi: 10.3389/fpls.2018.00980 PMC6092734 30135691

[B2] Bidzinski P. Ballini E. Ducasse A. Michel C. Zuluaga P. Genga A.. (2016). Transcriptional basis of drought-induced susceptibility to the rice blast fungus *Magnaporthe oryzae* . Front. Plant Sci. 7. doi: 10.3389/fpls.2016.01558 PMC5081564 27833621

[B3] Castroagudin V. L. Moreira S. I. Pereira D. A. Moreira S. S. Brunner P. C. Maciel J. L.. (2016). *Pyricularia graminis-tritici*, a new *Pyricularia* species causing wheat blast. Persoonia 37, 199–216. doi: 10.3767/003158516X692149 28232765 PMC5315288

[B4] Ceresini P. C. Castroagudin V. L. Rodrigues F. A. Rios J. A. Eduardo Aucique-Perez C. Moreira S. I.. (2018). Wheat blast: Past, present, and future. Annu. Rev. Phytopathol. 56, 427–456. doi: 10.1146/annurev-phyto-080417-050036 29975608

[B5] Chi M. H. Park S. Y. Kim S. Lee Y. H. (2009). A novel pathogenicity gene is required in the rice blast fungus to suppress the basal defenses of the host. PloS Pathog. 5, e1000401. doi: 10.1371/journal.ppat.1000401 19390617 PMC2668191

[B6] Christodoulou M. S. Pinna C. Ghosh S. Princiotto S. Sacchi F. Brunetti B.. (2024). Natural and nature-inspired catechol siderophores: A promising strategy for rice blast management. J. Agric. Food Chem. 72, 22439–22450. doi: 10.1021/acs.jafc.4c02909 39365249

[B7] Cimini S. Locato V. Giacinti V. Molinari M. De Gara L. (2022). A multifactorial regulation of glutathione metabolism behind salt tolerance in rice. Antioxidants (Basel) 11, 1114. doi: 10.3390/antiox11061114 35740011 PMC9219684

[B8] Cruz C. D. Valent B. (2017). Wheat blast disease: danger on the move. Trop. Plant Pathol. 42, 210–222. doi: 10.1007/s40858-017-0159-z

[B9] Dangol S. Chen Y. Hwang B. K. Jwa N.-S. (2019). Iron- and reactive oxygen species-dependent ferroptotic cell death in rice-*Magnaporthe oryzae* interactions. Plant Cell 31, 189–209. doi: 10.1105/tpc.18.00535 30563847 PMC6391706

[B10] Dean R. Van Kan J. A. Pretorius Z. A. Hammond-Kosack K. E. Di Pietro A. Spanu P. D.. (2012). The Top 10 fungal pathogens in molecular plant pathology. Mol. Plant Pathol. 13, 414–430. doi: 10.1111/j.1364-3703.2011.00783.x 22471698 PMC6638784

[B11] Dixon S. J. Lemberg K. M. Lamprecht M. R. Skouta R. Zaitsev E. M. Gleason C. E.. (2012). Ferroptosis: an iron-dependent form of nonapoptotic cell death. Cell 149, 1060–1072. doi: 10.1016/j.cell.2012.03.042 22632970 PMC3367386

[B12] Dludla P. V. Ziqubu K. Mabhida S. E. Mazibuko-Mbeje S. E. Hanser S. Nkambule B. B.. (2023). Dietary supplements potentially target plasma glutathione levels to improve cardiometabolic health in patients with diabetes mellitus: A systematic review of randomized clinical trials. Nutrients 15, 944. doi: 10.3390/nu15040944 36839303 PMC9966974

[B13] Egan M. J. Wang Z. Y. Jones M. A. Smirnoff N. Talbot N. J. (2007). Generation of reactive oxygen species by fungal NADPH oxidases is required for rice blast disease. Proc. Natl. Acad. Sci. U.S.A. 104, 11772–11777. doi: 10.1073/pnas.0700574104 17600089 PMC1913907

[B14] Fernandez J. Wilson R. A. (2014). Characterizing roles for the glutathione reductase, thioredoxin reductase and thioredoxin peroxidase-encoding genes of *Magnaporthe oryzae* during rice blast disease. PloS One 9, e87300. doi: 10.1371/journal.pone.0087300 24475267 PMC3901745

[B15] Fisher M. C. Hawkins N. J. Sanglard D. Gurr S. J. (2018). Worldwide emergence of resistance to antifungal drugs challenges human health and food security. Science 360, 739–742. doi: 10.1126/science.aap7999 29773744

[B16] Friedmann Angeli J. P. Schneider M. Proneth B. Tyurina Y. Y. Tyurin V. A. Hammond V. J.. (2014). Inactivation of the ferroptosis regulator Gpx4 triggers acute renal failure in mice. Nat. Cell Biol. 16, 1180–1191. doi: 10.1038/ncb3064 25402683 PMC4894846

[B17] Ge C. Zhang S. Mu H. Zheng S. Tan Z. Huang X.. (2021). Emerging mechanisms and disease implications of ferroptosis: Potential applications of natural products. Front. Cell Dev. Biol. 9. doi: 10.3389/fcell.2021.774957 PMC8804219 35118067

[B18] Goufo P. Trindade H. (2017). Factors influencing antioxidant compounds in rice. Crit. Rev. Food Sci. Nutr. 57, 893–922. doi: 10.1080/10408398.2014.922046 25897468

[B19] Hasanuzzaman M. Nahar K. Anee T. I. Fujita M. (2017). Glutathione in plants: biosynthesis and physiological role in environmental stress tolerance. Physiol. Mol. Biol. Plants 23, 249–268. doi: 10.1007/s12298-017-0422-2 28461715 PMC5391355

[B20] Hollomon W. D. (2015). Fungicide resistance: facing the challenge - a review. Plant Prot. Sci. 51, 170–176. doi: 10.17221/42/2015-PPS

[B21] Hu Y. Zhang J. Kong W. Zhao G. Yang M. (2017). Mechanisms of antifungal and anti-aflatoxigenic properties of essential oil derived from turmeric (*Curcuma longa* L.) on *Aspergillus flavus* . Food Chem. 220, 1–8. doi: 10.1016/j.foodchem.2016.09.179 27855875

[B22] Huang K. Czymmek K. J. Caplan J. L. Sweigard J. A. Donofrio N. M. (2011). HYR1-mediated detoxification of reactive oxygen species is required for full virulence in the rice blast fungus. PloS Pathog. 7, e1001335. doi: 10.1371/journal.ppat.1001335 21533213 PMC3077360

[B23] Ikeda K.-i. Uchihashi K. Okuda I. Xiang Z. Nakayashiki H. (2024). Specific detection of *Pyricularia oryzae* pathotype *Triticum* using qPCR and LAMP methods. J. Gen. Plant Pathol. 90, 82–94. doi: 10.1007/s10327-023-01162-0

[B24] Islam T. Ansary M. W. R. Rahman M. M. (2023). “ *Magnaporthe oryzae* and its pathotypes: A potential plant pandemic threat to global food security,” in Plant relationships: fungal-plant interactions. Eds. Scott B. Mesarich C. (Springer International Publishing, Cham), 425–462.

[B25] Kaku H. Nishizawa Y. Ishii-Minami N. Akimoto-Tomiyama C. Dohmae N. Takio K.. (2006). Plant cells recognize chitin fragments for defense signaling through a plasma membrane receptor. Proc. Natl. Acad. Sci. U.S.A. 103, 11086–11091. doi: 10.1073/pnas.0508882103 16829581 PMC1636686

[B26] Kato T. Tanabe S. Nishimura M. Ohtake Y. Nishizawa Y. Shimizu T.. (2009). Differential responses of rice to inoculation with wild-type and non-pathogenic mutants of *Magnaporthe oryzae* . Plant Mol. Biol. 70, 617–625. doi: 10.1007/s11103-009-9495-9 19418231

[B27] Kou Y. Qiu J. Tao Z. (2019). Every coin has two sides: reactive oxygen species during rice–*Magnaporthe oryzae* interaction. Int. J. Mol. Sci. 20, 1191. doi: 10.3390/ijms20051191 30857220 PMC6429160

[B28] Liang M. Ye H. Shen Q. Jiang X. Cui G. Gu W.. (2021). Tangeretin inhibits fungal ferroptosis to suppress rice blast. J. Integr. Plant Biol. 63, 2136–2149. doi: 10.1111/jipb.13175 34570416

[B29] Liu Q. Long R. Lin C. Bi X. Liang Z. Deng Y. Z. (2024). Phosphatidylethanolamines link ferroptosis and autophagy during appressorium formation of rice blast fungus. Mol. Plant Pathol. 25, e13489. doi: 10.1111/mpp.13489 38956897 PMC11219472

[B30] Liu X. Zhang Z. (2022). A double-edged sword: reactive oxygen species (ROS) during the rice blast fungus and host interaction. FEBS J. 289, 5505–5515. doi: 10.1111/febs.16171 34453409

[B31] Mendoza-Sarmiento D. Mistades E. V. Hill A. M. (2023). Effect of pigmented rice consumption on cardiometabolic risk factors: A systematic review of randomized controlled trials. Curr. Nutr. Rep. 12, 797–812. doi: 10.1007/s13668-023-00496-7 37676476 PMC10766681

[B32] Miotto G. Rossetto M. Di Paolo M. L. Orian L. Venerando R. Roveri A.. (2020). Insight into the mechanism of ferroptosis inhibition by ferrostatin-1. Redox Biol. 28, 101328. doi: 10.1016/j.redox.2019.101328 31574461 PMC6812032

[B33] Moin A. T. Robin T. B. Patil R. B. Rani N. A. Prome A. A. Sakif T. I.. (2024). Antifungal plant flavonoids identified in silico with potential to control rice blast disease caused by *Magnaporthe oryzae* . PloS One 19, e0301519. doi: 10.1371/journal.pone.0301519 38578751 PMC10997076

[B34] Molina-Hernandez J. B. Capelli F. Laurita R. Tappi S. Laika J. Gioia L.. (2022). A comparative study on the antifungal efficacy of cold atmospheric plasma at low and high surface density on *Aspergillus chevalieri* and mechanisms of action. Innov. Food Sci. Emerg. Technol. 82, 103194. doi: 10.1016/j.ifset.2022.103194

[B35] Muthayya S. Sugimoto J. D. Montgomery S. Maberly G. F. (2014). An overview of global rice production, supply, trade, and consumption. Ann. N.Y. Acad. Sci. 1324, 7–14. doi: 10.1111/nyas.12540 25224455

[B36] Nalley L. Tsiboe F. Durand-Morat A. Shew A. Thoma G. (2016). Economic and environmental impact of rice blast pathogen (*Magnaporthe oryzae*) alleviation in the United States. PloS One 11, e0167295. doi: 10.1371/journal.pone.0167295 27907101 PMC5131998

[B37] Onaga G. Wydra K. D. Koopmann B. Sere Y. von Tiedemann A. (2017). Elevated temperature increases in planta expression levels of virulence related genes in *Magnaporthe oryzae* and compromises resistance in *Oryza sativa* cv. *Nipponbare* . Funct. Plant Biol. 44, 358–371. doi: 10.1071/FP16151 32480570

[B38] Pang Y. Ahmed S. Xu Y. Beta T. Zhu Z. Shao Y.. (2018). Bound phenolic compounds and antioxidant properties of whole grain and bran of white, red and black rice. Food Chem. 240, 212–221. doi: 10.1016/j.foodchem.2017.07.095 28946264

[B39] Qiu J. Liu Z. Xie J. Lan B. Shen Z. Shi H.. (2022). Dual impact of ambient humidity on the virulence of *Magnaporthe oryzae* and basal resistance in rice. Plant Cell Environ. 45, 3399–3411. doi: 10.1111/pce.14452 36175003

[B40] Quideau S. Deffieux D. Douat-Casassus C. Pouysegu L. (2011). Plant polyphenols: chemical properties, biological activities, and synthesis. Angew. Chem. Int. Ed. Engl. 50, 586–621. doi: 10.1002/anie.201000044 21226137

[B41] Riegman M. Sagie L. Galed C. Levin T. Steinberg N. Dixon S. J.. (2020). Ferroptosis occurs through an osmotic mechanism and propagates independently of cell rupture. Nat. Cell Biol. 22, 1042–1048. doi: 10.1038/s41556-020-0565-1 32868903 PMC7644276

[B42] Rizzardi N. Pezzolesi L. Samori C. Senese F. Zalambani C. Pitacco W.. (2022). Natural astaxanthin is a green antioxidant able to counteract lipid peroxidation and ferroptotic cell death. Int. J. Mol. Sci. 23, 15137. doi: 10.3390/ijms232315137 36499464 PMC9737268

[B43] Samalova M. Meyer A. J. Gurr S. J. Fricker M. D. (2014). Robust anti-oxidant defences in the rice blast fungus *Magnaporthe oryzae* confer tolerance to the host oxidative burst. New Phytol. 201, 556–573. doi: 10.1111/nph.12530 24117971

[B44] Scarpellini C. Klejborowska G. Lanthier C. Hassannia B. Vanden Berghe T. Augustyns K. (2023). Beyond ferrostatin-1: a comprehensive review of ferroptosis inhibitors. Trends Pharmacol. Sci. 44, 902–916. doi: 10.1016/j.tips.2023.08.012 37770317

[B45] Scheuermann K. K. Vieira Raimondi J. Marschalek R. de Andrade A. Wickert E. (2012). “ *Magnaporthe oryzae* genetic diversity and its outcomes on the search for durable resistance,” in The molecular basis of plant genetic diversity. Ed. Mahmut C. (IntechOpen, Rijeka), 331–356.

[B46] Shalaby S. Horwitz B. A. (2015). Plant phenolic compounds and oxidative stress: integrated signals in fungal-plant interactions. Curr. Genet. 61, 347–357. doi: 10.1007/s00294-014-0458-6 25407462

[B47] Shen Q. Liang M. Yang F. Deng Y. Z. Naqvi N. I. (2020). Ferroptosis contributes to developmental cell death in rice blast. New Phytol. 227, 1831–1846. doi: 10.1111/nph.16636 32367535

[B48] Shen Q. Naqvi N. I. (2021). Ferroptosis and microbial pathogenesis. PloS Pathog. 17, e1009298. doi: 10.1371/journal.ppat.1009298 33662044 PMC7932112

[B49] Shen Q. Naqvi N. I. (2024). The ferroptosis landscape of biotic interactions in plants. Curr. Opin. Plant Biol. 77, 102499. doi: 10.1016/j.pbi.2023.102499 38142619

[B50] Shen Q. Yang F. Naqvi N. I. (2023). A mitochondrial regulon for developmental ferroptosis in rice blast. bioRxiv 2023, 2005.2017.541075. doi: 10.1101/2023.05.17.541075

[B51] Sies H. Berndt C. Jones D. P. (2017). Oxidative stress. Annu. Rev. Biochem. 86, 715–748. doi: 10.1146/annurev-biochem-061516-045037 28441057

[B52] Singh B. K. Delgado-Baquerizo M. Egidi E. Guirado E. Leach J. E. Liu H.. (2023). Climate change impacts on plant pathogens, food security and paths forward. Nat. Rev. Microbiol. 21, 640–656. doi: 10.1038/s41579-023-00900-7 37131070 PMC10153038

[B53] Singh R. Maurya S. (2021). “Blast disease of rice: Evolution and adaptation in context of changing climate,” in Blast disease of cereal crops: evolution and adaptation in context of climate change. Eds. Nayaka S. C. Hosahatti R. Prakash G. Satyavathi C. T. Sharma R. (Springer International Publishing, Cham), 125–133.

[B54] Stockwell B. R. Friedmann Angeli J. P. Bayir H. Bush A. I. Conrad M. Dixon S. J.. (2017). Ferroptosis: A regulated cell death nexus linking metabolism, redox biology, and disease. Cell 171, 273–285. doi: 10.1016/j.cell.2017.09.021 28985560 PMC5685180

[B55] Valent B. (2021). “The impact of blast disease: Past, present, and future,” in Magnaporthe oryzae: methods and protocols. Ed. Jacob S. (Springer US, New York, NY), 1–18. 10.1007/978-1-0716-1613-0_1 34236673

[B56] Wilson R. A. (2021). Magnaporthe oryzae. Trends Microbiol. 29, 663–664. doi: 10.1016/j.tim.2021.03.019 33926783

[B57] Yang X. Yan S. Li G. Li Y. Li J. Cui Z.. (2024). Rice-*Magnaporthe oryzae* interactions in resistant and susceptible rice cultivars under panicle blast infection based on defense-related enzyme activities and metabolomics. PloS One 19, e0299999. doi: 10.1371/journal.pone.0299999 38451992 PMC10919634

[B58] Zhang R. Kroemer G. Tang D. (2024). Lipid-derived radical-trapping antioxidants suppress ferroptosis. Life Metab. 3, loae008. doi: 10.1093/lifemeta/loae008 38523816 PMC10960586

[B59] Zhang S. Hu R. Geng Y. Chen K. Wang L. Imam M. U. (2021). The regulatory effects and the signaling pathways of natural bioactive compounds on ferroptosis. Foods 10, 2952. doi: 10.3390/foods10122952 34945503 PMC8700948

