# Supplementary material for: Antioxidant-mediated suppression of ferroptosis in Pyricularia oryzae: a novel approach to rice blast management for sustainable rice production
Source: Front Plant Sci. 2024 Dec 20;15:1520688. doi: 10.3389/fpls.2024.1520688 (PMC11695299; doi:10.3389/fpls.2024.1520688)
Supplement: Supplementary file 1 [file Table1.docx]

Supplementary Material

**Supplementary Table 1.** Major classes of polyphenolic compounds (PCs) identified in rice and their roles in plant stress responses.

| PC Classes | Compound Types | Examples | Role in Stress | References |
| --- | --- | --- | --- | --- |
| Flavonoids | Flavones | Tricin | Antioxidant activity | Ajitha et al., 2012 |
|  |  | Apigenin *C*-glycosides schaftoside | Antioxidant and antifeedant activity | Stevenson et al., 1996; Yang et al., 2016 |
|  | Flavonols | Quercetin | Antioxidant activity | Goufo and Trindade, 2014 |
|  | Anthocyanins ^†^ | Cyanidin-3-glucoside, Peonidin-3-glucoside; Malvidin-3-glucoside | Antioxidant activity | Tamura et al., 2010; Goufo and Trindade, 2014 |
| Phenolic Acids | Hydroxybenzoic acids | *p*-Hydroxybenzoic acid; Protocatechuic acid; Vanillic acid; Syringic acid | Antioxidant and antimicrobial activity | Adom and Liu, 2002; Seal et al., 2004 |
|  | Hydroxycinnamic acids | Ferulic acid; *p*-Coumaric acid | Antioxidant and antimicrobial activity | Wang et al., 2018; Liu et al., 2022 |

^†^ present in colored rice varieties.

**References**

Adom, K.K., and Liu, R.H. (2002). Antioxidant activity of grains. *J. Agric. Food Chem.* 50, 6182-6187. doi: 10.1021/jf0205099

Ajitha, M.J., Mohanlal, S., Suresh, C.H., and Jayalekshmy, A. (2012). DPPH radical scavenging activity of tricin and its conjugates isolated from "Njavara" rice bran: a density functional theory study. *J. Agric. Food Chem.* 60, 3693-3639. doi: 10.1021/jf204826e

Goufo, P., and Trindade, H. (2014). Rice antioxidants: phenolic acids, flavonoids, anthocyanins, proanthocyanidins, tocopherols, tocotrienols, γ-oryzanol, and phytic acid. *Food Sci. Nutr.* 2, 75-104. doi: 10.1002/fsn3.86

Liu, S., Jiang, J., Ma, Z., Xiao, M., Yang, L., Tian, B., et al. (2022). The role of hydroxycinnamic acid amide pathway in plant immunity. *Front. Plant Sci.* 13, 922119. doi: 10.3389/fpls.2022.922119

Seal, A.N., Pratley, J.E., Haig, T., and An, M. (2004). Identification and quantitation of compounds in a series of allelopathic and non-allelopathic rice root exudates. *J. Chem. Ecol.* 30, 1647-1662. doi: 10.1023/B:JOEC.0000042074.96036.14

Stevenson, P.C., Kimmins, F.M., Grayer, R.J., and Raveendranath, S. (1996). "Schaftosides from rice phloem as feeding inhibitors and resistance factors to brown planthoppers, *Nilaparvata lugens*," in *Proceedings of the 9th International Symposium on Insect-Plant Relationships,* eds. E. Städler, M. Rowell-Rahier & R. Bauer (Dordrecht: Springer Netherlands), 246-249.

Tamura, S., Yan, K., Shimoda, H., and Murakami, N. (2010). Anthocyanins from *Oryza sativa* L. subsp. *indica*. *Biochem. Syst. Ecol.* 38, 438-440. doi: 10.1016/j.bse.2010.01.017

Wang, W., Li, Y., Dang, P., Zhao, S., Lai, D., and Zhou, L. (2018). Rice secondary metabolites: Structures, roles, biosynthesis, and metabolic regulation. *Molecules* 23, 3098. doi: 10.3390/molecules23123098

Yang, Z., Nakabayashi, R., Mori, T., Takamatsu, S., Kitanaka, S., and Saito, K. (2016). Metabolome analysis of *Oryza sativa* (Rice) using liquid chromatography-mass spectrometry for characterizing organ specificity of flavonoids with anti-inflammatory and anti-oxidant activity. *Chem. Pharm. Bull. (Tokyo)* 64, 952-956. doi: 10.1248/cpb.c16-00180
